# Assignment to More Senior Pediatric Cardiac Surgeons Following Socioeconomic Status Does Not Affect Operative Mortality

**DOI:** 10.1016/j.jacadv.2025.102018

**Published:** 2025-07-22

**Authors:** Mitchell C. Haverty, Rittal Mehta, In Hye Park, David Wessel, Yves d’Udekem, Jennifer H. Klein

**Affiliations:** a Division of Cardiovascular Surgery, Department of Pediatrics, Children's National Hospital, Washington, District of Columbia, USA; b Division of Cardiology, Department of Pediatrics, Children's National Hospital, Washington, District of Columbia, USA

**Keywords:** cardiovascular surgery, congenital, pediatrics, seniority, socioeconomics

**What is the clinical question being addressed?**

Is a difference in distribution of senior surgeons responsible for disparities in outcomes after pediatric cardiac surgery?

**What is the main finding?**

Socioeconomically advantaged patients are more often directed to senior surgeons, although this is not responsible for improved operative mortality.

Surgeon case experience is regularly associated with better postoperative outcomes in a cardiac surgery setting.^1^ Despite growing consensus, the impact of an individual congenital cardiac surgeon’s level of seniority on surgical outcomes has not been widely reported. While studies have explored the impact of surgeon experience on outcomes after congenital heart surgery,^2^ the association between socioeconomic status—a known social determinant of health in pediatric heart surgery outcomes—and surgeon seniority remains underexplored.^3^ Given that socioeconomic status is associated with worse short-term and long-term outcomes after pediatric cardiac surgery,^3^^,^^4^ we investigated whether socioeconomic status is associated with differences in surgeon assignment and therefore a potential driver of disparities in surgical outcomes.

## Methods

A single-center retrospective analysis included individuals who underwent congenital cardiac surgery over a 16-year period (January 2007-2023). Individuals with invalid addresses and those residing outside of the Washington, DC, metro area (as defined by the U.S. Office of Management and Budget) were excluded from the study. Only initial index surgeries were included in the analysis. Minor, nondefect-correcting surgeries were excluded (isolated pacemakers and ventricular assist device implantations, patent ductus arteriosus closures, and pericardial drainages).

A review of the electronic medical record collected clinical and demographic data. The patient’s neighborhood socioeconomic background was determined using the Child Opportunity Index (COI), a composite index that assesses the health, environmental, educational, social, and economic factors of a neighborhood and assigns neighborhoods a quintile rank.^5^ Neighborhoods in the lowest 2 COI quintiles were grouped and considered “disadvantaged,” while neighborhoods in the highest 2 COI quintiles were grouped and considered “advantaged.” Neighborhoods in the middle “moderate” quintile were ungrouped. A total of 13 surgeons (including 2 chiefs considered the senior surgeons) operated within the study period. Year as a predictor was investigated, though it did not improve model fit. For consistency, nonchief surgeons were classified as junior, though years of practice may have increased experience throughout the study period. All surgeons were eligible to take any case (with equivalent insurance coverages and call schedules). Except for emergencies presented on-call, surgeon assignment was determined by committee with the senior surgeon as the final decision maker, though surgeons scrubbed in to help as needed. Operative mortality was defined using the Society of Thoracic Surgeons definition as death within 30 days of operation or during hospitalization.

Bivariable and multivariable logistic regression models assessed the association among patient characteristics and operative mortality, with results presented as the OR with a 95% CI. The design of the study was approved by the ethics committee, and a waiver of consent for data acquisition was issued from the institutional review board.

## Results

### Characteristics

Of the 3,649 patients identified at our center, 2,775 met inclusion criteria. The plurality of the cohort were male (54.8%, n = 1,521), operated on by nonsenior surgeons (63.1%, n = 1,750), and were from disadvantaged neighborhoods (48.4%, n = 1,344). Most operations required cardiopulmonary bypass (n = 2,413, 87.0%) and were The Society of Thoracic Surgeons (STS)-European Association for Cardio-Thoracic Surgery (EACTS) Congenital Heart Surgery category 1 (n = 1,468, 52.9%). Compared to those from moderate and advantaged neighborhoods, patients from socioeconomically disadvantaged neighborhoods had lower birthweight (*P* = 0.050), higher rates of genetic abnormalities (*P* = 0.012), and a lower rate of prenatal diagnosis (*P* = 0.035). Individuals from disadvantaged neighborhoods also had longer median (Q1-Q3) cardiopulmonary bypass times (105 [78-142] vs 94 [71-127] vs 93 [69-131] minutes; *P* < 0.001) and longer median (Q1-Q3) cross clamp times compared to moderate and advantaged individuals (54 [39-80] vs 50 [36-74] vs 51 [37-76] minutes; *P* < 0.001). Individuals from disadvantaged neighborhoods also had a higher rate of operations performed in the neonatal period (n = 371 [27.6%] vs n = 132 [23.1%] vs n = 211 [25.6%]; *P* = 0.042) and more frequent urgent procedures (n = 409 [30.4%] vs n = 146 [25.5%] vs n = 194 [22.6%]; *P* = 0.005). The Society of Thoracic Surgeons (STS)-European Association for Cardio-Thoracic Surgery (EACTS) Congenital Heart Surgery category 5 distribution differed among those from disadvantaged, moderate, and advantaged neighborhoods (n = 84 [6.3%] vs 28 [4.9%] vs 43 [5.0%]; *P* = 0.019), though likely not to a clinically relevant level. Individuals from disadvantaged neighborhoods, as compared to those from moderate and advantaged neighborhoods, were assigned to junior surgeons more frequently (n = 952 [70.8%] vs n = 350 [61.2%] vs n = 448 [52.2%]; *P* < 0.001). Compared to moderate and advantaged cohorts, disadvantaged individuals had higher rates of major complications (n = 191 [14.2%] vs 48 [8.4%] vs 92 [10.7%]; *P* < 0.001), longer median (Q1-Q3) postoperative length of stay (9 [5-22] vs 6 [4-16] vs 6 [4-15] days; *P* < 0.001), and higher operative mortality (n = 88 [6.5%] vs 22 [3.8%] vs 37 [4.3%]; *P* = 0.016).

Additional analysis found that senior, compared to junior surgeons, had no difference in operative mortality (n = 57 [5.6%] vs 90 [5.1%]; *P* = 0.700), but had shorter median (Q1-Q3) cardiopulmonary bypass times (83 [66-133] vs 111 [83-146] minutes; *P* < 0.001) and cross clamp times (46 [34-64] vs 61 [42-84] minutes; *P* < 0.001). Senior surgeons had fewer The Society of Thoracic Surgeons (STS)-European Association for Cardio-Thoracic Surgery (EACTS) Congenital Heart Surgery category 5 cases (53 [5.2%] vs 102 [5.8%]; *P* < 0.001), though this difference likely did not reach clinical relevance.

### Outcomes

An unadjusted bivariable analysis of operative mortality found that the seniority of the surgeon assigned to the case did not result in increased odds of operative mortality (OR: 0.92, 95% CI: 0.66-1.30; *P* = 0.640). After adjusting for confounders by multivariable analysis, the only independent predictors for mortality were urgent (OR: 1.66, *P* = 0.018) or emergent status (OR: 7.11, *P* < 0.001) compared to elective, lower weight at surgery (OR: 0.40, *P* < 0.001), prematurity (OR: 2.07, *P* = 0.001), genetic abnormalities (OR: 1.62, *P* = 0.023), or higher The Society of Thoracic Surgeons (STS)-European Association for Cardio-Thoracic Surgery (EACTS) Congenital Heart Surgery category as compared to category 1 (The Society of Thoracic Surgeons (STS)-European Association for Cardio-Thoracic Surgery (EACTS) Congenital Heart Surgery 2: *P* < 0.001, The Society of Thoracic Surgeons (STS)-European Association for Cardio-Thoracic Surgery (EACTS) Congenital Heart Surgery 3: *P* = 0.008, The Society of Thoracic Surgeons (STS)-European Association for Cardio-Thoracic Surgery (EACTS) Congenital Heart Surgery 4: *P* = 0.001, The Society of Thoracic Surgeons (STS)-European Association for Cardio-Thoracic Surgery (EACTS) Congenital Heart Surgery 5: *P* < 0.001) to be associated with higher risk of operative mortality (Table 1).

**Table 1:** Multivariable Analysis Describing the Predictive Factors of Operative Mortality and Major Complications

	OR (95% CI)	*P* Value
Operative mortality		
Status		
Elective vs emergent	7.11 (3.008, 16.810)	**<0.001**
Elective vs urgent	1.66 (1.092, 2.531)	**0.018**
Weight at surgery (kg)	0.40 (0.260, 0.613)	**<0.001**
The Society of Thoracic Surgeons (STS)-European Association for Cardio-Thoracic Surgery (EACTS) Congenital Heart Surgery category		
2 vs 1	2.60 (1.280, 5.287)	**<0.001**
3 vs 1	6.41 (3.251, 12.626)	**0.008**
4 vs 1	7.83 (3.903, 15.716)	**<0.001**
5 vs 1	15.95 (8.041, 31.628)	**<0.001**
Premature birth		
Yes vs no	2.07 (1.332, 3.216)	**0.001**
Genetic abnormality		
Yes vs no	1.62 (1.071, 2.453)	**0.023**
Major complications		
Senior surgeon assignment	0.721 (0.537, 0.967)	**0.029**
Status		
Elective vs emergent	0.294 (0.135, 0.640)	**<0.001**
The Society of Thoracic Surgeons (STS)-European Association for Cardio-Thoracic Surgery (EACTS) Congenital Heart Surgery category		
2 vs 1	2.12 (1.445, 3.109)	**0.001**
3 vs 1	3.92 (2.618, 5.877)	**<0.001**
4 vs 1	2.77 (1.727, 4.451)	**<0.001**
5 vs 1	8.86 (5.506, 14.244)	**<0.001**
Premature birth		
Yes vs no	1.95 (1.42, 2.674)	**<0.001**
Genetic abnormality		
Yes vs no	1.97 (1.467, 2.674)	**<0.001**

**Bold** values are statistically significant (*p* values <0.05).

## Discussion

Patients from higher socioeconomic background are more often directed to the care of the most senior surgeon, an obvious fact that has not been very well described. Though, like others, we have previously demonstrated that individuals from socioeconomically disadvantaged backgrounds undergoing cardiac surgery are at greater risk of operative mortality,^3^ our current study suggests that this higher mortality is not mediated by the disparity in the seniority of surgeon assigned to their case. The findings confirm data reported by Anderson et al showing lower surgeon experience was not associated with increased risk of operative mortality.^2^ Instead, clinical variables such as weight at surgery, prematurity, and the presence of genetic abnormalities should be examined as mediators for the higher operative mortality rates observed in disadvantaged communities.

## Conclusions

Patients from neighborhoods of higher socioeconomic status were more likely to receive treatment from a senior surgeon. However, assignment of a senior surgeon had no impact on operative mortality.

## Funding support and author disclosures

Dr d’Udekem is a consultant for Bayer Pharmaceuticals. All other authors have reported that they have no relationships relevant to the contents of this paper to disclose.
